# Substrate topographies modulate the secretory activity of human bone marrow mesenchymal stem cells

**DOI:** 10.1186/s13287-023-03450-0

**Published:** 2023-08-21

**Authors:** Heizel Rosado-Galindo, Maribella Domenech

**Affiliations:** 1 Bioengineering Program, University of Puerto Rico-Mayagüez, Road 108, KM 1.1., Mayagüez, PR 00680 USA; 2 Department of Chemical Engineering, University of Puerto Rico-Mayagüez, Road 108, KM 1.1., Mayagüez, PR 00680 USA

**Keywords:** Topography, Mesenchymal stem cells, Immunosuppression, Secretome

## Abstract

**Background:**

Mesenchymal stem cells (MSCs) secrete a diversity of factors with broad therapeutic potential, yet current culture methods limit potency outcomes. In this study, we used topographical cues on polystyrene films to investigate their impact on the secretory profile and potency of bone marrow-derived MSCs (hBM-MSCs). hBM-MSCs from four donors were cultured on topographic substrates depicting defined roughness, curvature, grooves and various levels of wettability.

**Methods:**

The topographical PS-based array was developed using razor printing, polishing and plasma treatment methods. hBM-MSCs from four donors were purchased from RoosterBio and used in co-culture with peripheral blood mononuclear cells (PBMCs) from Cell Applications Inc. in an immunopotency assay to measure immunosuppressive capacity. Cells were cultured on low serum (2%) for 24–48 h prior to analysis. Image-based analysis was used for cell quantification and morphology assessment. Metabolic activity of BM-hMSCs was measured as the mitochondrial oxygen consumption rate using an extracellular flux analyzer. Conditioned media samples of BM-hMSCs were used to quantify secreted factors, and the data were analyzed using R statistics. Enriched bioprocesses were identify using the Gene Ontology tool *enrichGO* from the *clusterprofiler.* One-way and two-way ANOVAs were carried out to identify significant changes between the conditions. Results were deemed statistically significant for combined *P* < 0.05 for at least three independent experiments.

**Results:**

Cell viability was not significantly affected in the topographical substrates, and cell elongation was enhanced at least twofold in microgrooves and surfaces with a low contact angle. Increased cell elongation correlated with a metabolic shift from oxidative phosphorylation to a glycolytic state which is indicative of a high-energy state. Differential protein expression and gene ontology analyses identified bioprocesses enriched across donors associated with immune modulation and tissue regeneration. The growth of peripheral blood mononuclear cells (PBMCs) was suppressed in hBM-MSCs co-cultures, confirming enhanced immunosuppressive potency. YAP/TAZ levels were found to be reduced on these topographies confirming a mechanosensing effect on cells and suggesting a potential role in the immunomodulatory function of hMSCs.

**Conclusions:**

This work demonstrates the potential of topographical cues as a culture strategy to improve the secretory capacity and enrich for an immunomodulatory phenotype in hBM-MSCs.

**Supplementary Information:**

The online version contains supplementary material available at 10.1186/s13287-023-03450-0.

## Background

Mesenchymal stem cells (MSCs) are multipotent cells that have become a promising therapeutic agent due to their self-renewal capability, differentiation into various cell lineages and immune regulation associated with their secretion of factors [1]. Much of the research efforts on MSCs have focused on the use of autologous or allogeneic infusion of MSCs to treat a wide range of conditions (e.g., heart [2, 3], autoimmune diseases [4–6], wounds [7, 8] and neurodegenerative diseases [9]). However, it has been demonstrated that the main therapeutic efficacy of MSCs is done through paracrine stimulation via the secretion of biomolecules into the affected area [10]. The secretome of human MSCs is composed of cytokines, growth factors, extracellular vesicles (EV) and noncoding RNAs [11, 12]. It has been studied for more than two decades and provides an alternative over cell therapy in terms of safety and clinical use. Despite the value of MSC secretome, no secretome-based therapy has been approved by the US Food Drug Administration (FDA). This is mainly due to the need for in depth characterization of its composition, heterogeneity of the harvested product dependent on donor, tissue source and culture conditions and scalability of production [11]. Therefore, understanding which molecules secreted by MSCs are of therapeutic value and generating strategies to enhance the secretory capacity of MSCs in a cost-effective manner are of utmost importance to advance the field.

In order to improve therapeutic outcomes and avoid inconsistent responses, researchers have proposed various ways to manipulate the MSCs secretome. The main approaches used to modulate the secretory profile of MSCs include priming and genetic modification [11, 12]. MSCs priming can be performed by treating the cells with inflammatory cytokines, pharmacological drugs, in hypoxia conditions and by modification of cell culture conditions and use of biomaterials [13]. Priming MSCs with inflammatory cytokines (e.g., IFN-ɣ, IL-17, TNF-ɑ) enhances the immunosuppressive capacity of the cells and increases the secretion of anti-inflammatory factors [14]. In a similar manner, it has been shown that MSCs cultured under hypoxia conditions have enhanced angiogenic and regenerative secretome [15, 16] and MSCs cultured in 3D bioscaffolds that mimic in vivo conditions have increased immunomodulatory potential and stemness [17]. However, priming approaches still have some limitations: they are expensive (e.g., use of recombinant cytokines) and cells from different sources show variable anti-inflammatory responses upon priming and increased immunogenicity [12]. Genetic modification of MSCs to overexpress or silence specific genes is a technique that has proved very successful as well by enhancing cell immunomodulatory, anti-inflammatory, regenerative and anti-apoptotic potency [18]. Yet, no standard protocol has been established for genetic modification of MSCs which implies increased variability in cell responses. Also, potential mutagenicity and immunogenicity of the viral vector can represent a safety concern [19].

One way to overcome these limitations can be using physical cues of the microenvironment. It is well known that substrate topography is a major regulator of MSCs behavior [20, 21], mostly influencing cytoskeleton organization [22], cell differentiation [23–25] and gene expression [26]. For example, Yang and collaborators demonstrated in their studies with hydroxyapatite scaffolds that optimal osteogenic differentiation was carried out at low levels of surface roughness [27]. Similarly, studies on the effect of spacing, orientation and diameter of micropore/nanorod patterned surfaces proved that straight patterns with relatively low spacing were more effective on MSCs proliferation and differentiation to osteogenic lineage for improved clinical performance of bone implants [28]. Our previous studies also demonstrated that polystyrene substrates with increased level of roughness enhanced the proliferation of adipose-derived MSCs and the expression of factors associated with proliferation, and thus, this physical stimulation could minimize the need for biochemical supplementation in cultures [29]. Likewise, surface topography has been shown to influence the behavior of other types of cells. For example, macrophage elongation in micro- and nanopatterned grooves modulated their polarization toward an anti-inflammatory phenotype [30]. Other studies on cancer have shown that anisotropic topographies promote the proliferation of cancer cells, an effect called mechanically induced dormancy [31].

While the generation of surface topography has allowed for the study of cell changes at the micro- and nanoscale level, these studies are carried out using microfabrication techniques that are not necessarily available to everyone. Techniques such as soft lithography [32], hot embossing [33] and micromilling [34] are often used to generate these topographies, yet most of them require high investment and technical expertise, and could produce toxic byproducts that can undermine the results. In this study, we used topographical features on polystyrene films to stimulate the secretion of MSCs. We fabricated microscale topographic substrates of defined roughness, curvature, grooves and various levels of wettability employing razor printing, sanding methods and plasma treatment. Our approach uses polystyrene as raw material, which is the gold standard for cell culture assays. It is non-biodegradable implying that the mechanical stimulation will not fade over time, as in for example hydroxyapatite [35] or Poly(ε-caprolactone) (PCL) [36]. Also, our micropatterns are easy to adapt into culture platforms for high-throughput analyses, are prototyped quickly, are affordable and do not require technical expertise to be generated. We demonstrated that topographical cues are an effective strategy to stimulate the secretory activity and immunosuppressive potency of MSCs.

## Methods

### Polystyrene topographical array fabrication

The topographical polystyrene-based (PS-based) array was developed using razor printing, oxygen plasma and sanding methods as described in our previous works (Additional file 1: Figure S1) [29, 37]. Topographies examined were roughness, curvature and wettability on flat PS films. Briefly, biaxially oriented, 0.19-mm-thick PS film (ST311190/3, Goodfellow) and medical-grade tape (ARCare 90,106) were used to generate the topographical sticker-like substrates. Grooves and spiral micropatterns were generated in the PS film using a cutting plotter (CE6000-40 Plus, Graphtec America, USA) equipped with a 0.9-mm-diameter and 60° angle Graphtec blade (CB09UA). PS films with surface roughness were generated using an in-house constructed device where a PS film sheet was placed between two plaques (the top plaque contained the sandpaper sheet incorporated) and manually pulled out of the device. The depth of the razor-printed micropatterns and surface roughness were measured and characterized using the Keyence 3D surface profiler (VK-X-1000, Keyence) and the VK analyzer software (Keyence Corporation), respectively. Levels of wettability were generated by controlling the amount of time the polystyrene films were exposed to oxygen plasma treatment (PE-50, Plasma Etch, Inc). Plasma-treated surfaces were characterized by measuring the surface contact angle of a water droplet (20µL), and the contact angle was analyzed using the ImageJ Contact Angle plug-in. The sticker-like substrates were taped to the bottom of the culture plates and sterilized using 3 cycles each consisting of 15 min exposure to UV light followed by a wash with 1X phosphate-buffered saline (PBS).

### Cell culture

Four healthy donors of human bone marrow-derived mesenchymal stem cells (hBM-MSCs) were purchased from Rooster Bio (MSC-003 lots: 00182, 310,271, 310,267 and 310,263) and used in the experiments with population doubling levels (PDL) between 13.2 and 15. The donors were derived from two females and two males, with ages ranging from 19 to 26 years. Cells were expanded in DMEM high glucose (D6429, Sigma) supplemented with 10% heat-inactivated fetal bovine serum (F4135, Sigma) and 1% penicillin–streptomycin (P4333, Sigma) and maintained at 37℃ and 5% CO_2_ in a humidified incubator. Peripheral blood mononuclear cells (PBMCs) were purchased from Cell Applications, Inc. (690 PB-100a) and maintained in RPMI-1640 medium (R8758, Sigma) supplemented with 10% heat-inactivated fetal bovine serum (F4135, Sigma), 1% penicillin–streptomycin (P4333, Sigma-Aldrich) and 1% nonessential amino acids—NEAA (M7145, Sigma).

### Cell viability

The viability of hBM-MSCs was assessed by performing the PrestoBlue™ cell viability assay (A13261, Invitrogen). Cells were seeded at a density of 9,000 cells/cm^2^ on each topography on a 96-well culture plate and their viability was measured after 5 days of culture in reduced serum media (2% FBS) using the protocol described by the manufacturer. Briefly, half of the media was removed and replaced with fresh 2% FBS media containing the PrestoBlue reagent (1:10) and the cells were incubated at 37 °C for 2 h. Lastly, the fluorescent intensity was measured using a Spark® multiplate reader (Tecan).

### Fluorescence staining

Morphology of the cells was measured by fluorescence staining of the cytoskeleton. hBM-MSCs were seeded at a density of 9,000 cells/cm^2^ on the topographical substrates attached to the bottom of 96-well culture plates and cultured for five days in reduced serum conditions (2% FBS). Afterward, cells were fixed for 15 min in 4% paraformaldehyde (sc-281692, Santa Cruz Biotechnology) and then permeabilized using 0.2% Triton X-100 (T8787, Sigma) in PBS for an additional 15 min at room temperature. Cytoskeleton staining was performed by incubating ActinRed™ 555 ReadyProbes reagent (R37112, Invitrogen) for 30 min, and cell nuclei were counterstained with Hoechst 33,342, 1:1000 dilution (H1399, Invitrogen) for 10 min at room temperature. Fluorescent images of the cells were acquired by taking 10X images using the Keyence BZ-X800 fluorescence microscope. Cell elongation factor was calculated as the ratio between the major axis and the minor axis of the cell. To do this, cells were manually traced in ImageJ software and the major and minor axes were calculated using the “fit ellipse” measurement in ImageJ. A hundred (100) cells per well were analyzed for each independent experiment.

*YAP/TAZ staining:* Cells were washed with PBS and fixed for 15 min in 4% paraformaldehyde, followed by permeabilization using 0.5% Triton X-100 (T8787, Sigma) in PBS for an additional 10 min. The cells were resuspended in blocking buffer 3% BSA in PBS + 0.1% Tween20 (P9416, Sigma) and incubated for 1 h. For staining, cells were incubated with anti-YAP/TAZ (D24E4, Cell Signaling) at a ratio of 1:250 in a 3% BSA in PBS + 0.1% Tween20 (P9416, Sigma) solution for 1 h at RT. Following that, cells were incubated with anti-rabbit Alexa Fluor 488 secondary antibody (ab150077, Abcam) at a ratio of 1:500 in a 3% BSA in PBS + 0.1% Tween20 solution for 1 h. Lastly, cells were counterstained with Hoechst 33,342 (1:1000 dilution) and washed three times with PBS. Fluorescent images were acquired by taking 10X images using the Keyence BZ-X800 fluorescence microscope. Fluorescent intensity was measured using ImageJ software (version 1.53a).

### Metabolic activity

The metabolic activity of hBM-MSCs cultured in the topographical substrates was assessed using Seahorse real-time cell metabolic analysis. Cells were seeded to confluence (10,000 cells/well) in the topographical surfaces attached to the bottom of XFe24 culture plates (03022–100, Agilent Technologies) and allowed to attach overnight. Then, culture media was changed to a reduced serum formulation containing 2% FBS and cells were cultured for 24 h. The Seahorse XF Mito Stress Test (103,010–100, Agilent Technologies) was performed to assess the mitochondrial function of the cells following manufacturer's protocol. Briefly, cells were washed twice with XFp Cell Mito Stress Assay medium which includes XF DMEM base medium with 2 mM glutamine, 10 mM glucose and 1 mM pyruvate (103,680–100, Agilent Technologies), and incubated for one hour at 37 °C without CO_2_. Oligomycin (1.5 µM), FCCP (1 µM) and rotenone/antimycin (0.5 µM) from the Mito Stress Kit were loaded into the plates, and oxygen consumption rate (OCR) was measured using the Seahorse XFe24 Extracellular Flux Analyzer (Seahorse Bioscience, USA). Cell nuclei were stained using Hoechst 33,342, and data were normalized by cell counts and analyzed using Wave software (Agilent).

### Exosome secretion

The isolation of hBM-MSCs exosomes was performed using the EXOSTEP™ kit (ExoS-25-C9, Immunostep). This kit is a bead-based assay that catches the exosomes in the culture media. Cells were seeded at a density of 25,000 cells/cm^2^ on the topographical substrates attached to a 96-well culture plate and allowed to attach overnight. Then, culture media was changed to a reduced serum formulation containing 2% FBS and cultured for 24 h. Afterward, the conditioned media was collected and CD63 + exosomes were detected following the manufacturer’s protocol. Briefly, conditioned media samples were pre-treated for direct exosome detection using two-step centrifugation. Then, the media samples were incubated overnight with CD63 + capture beads at room temperature and protected from the light. Afterward, captured exosomes were stained with anti-CD9 PE (Clone VJ1/20) and further analyzed using flow cytometry (BD Accuri™ C6 Plus). The mean fluorescent intensity in 10,000 events was used as a measure of the presence of exosomes in the samples.

### Cytokine expression

hBM-MSCs were seeded at a density of 9,000 cells/cm^2^ in the topographical substrates and allowed to attach overnight. Then, culture media was changed to a reduced serum formulation containing 2% FBS and cells were cultured for 5 days with media replacement at day 3. Indoleamine 2,3-dioxygenase (IDO) activity was performed by precipitating proteins from 100µL of cell supernatant with trichloroacetic acid (T6399, Sigma) 30% in 2:1 proportion. Then, samples were centrifuged at 950 g for 5 min and supernatant was collected and mixed with Ehrlich’s reagent (4-(dimethylamino) benzaldehyde) diluted in glacial acetic acid (ARK2183, Sigma) 20 mg/mL in a 1:1 proportion. Plate was incubated for 5 min, and the absorbance was measured at 490 nm using Spark® multiplate reader (Tecan). Absorbance reading was converted to kynurenine concentration using the equation of the standard calibration curve. Kynurenine concentrations were normalized to the number of cells. Quantification of the other cytokines—VEGF (ELH-VEGF, Raybiotech), TGFβ (ELH-TGFb1, Raybiotech), IL-10 (ELH-IL10, Raybiotech), TNF-ɑ (ELH-TNF-a, Raybiotech) and IL-1 (ELH-IL1a, Raybiotech)—was performed using enzyme-linked immunosorbent assay (ELISA), following manufacturer’s protocol. Briefly, 100 µL of conditioned media was incubated overnight at 4℃ to bind to the coated microplate. Then, wells were incubated for 1 h with the biotinylated antibody, followed by 45-min incubation with HRP-conjugated streptavidin and final TBM One-step substrate reagent incubation for 30 min. Stop solution was added at the end, and the absorbance was read at 450 nm using Spark® multiplate reader (Tecan). Absorbance reading was converted to the secreted factor concentration using the equation of the standard calibration curve. Cytokine’s concentrations were normalized to the number of cells per sample.

### Secretome analysis

hBM-MSCs were seeded in the topographical surfaces attached to 12-well plates at a density of 32,000 cells/well and allowed to attach overnight. Then, culture media was changed to a reduced serum formulation containing 2% FBS and cells were cultured for 5 days with media replacement on day 3. Afterward, conditioned media was collected, cells were lysed and samples were sent to RayBiotech for analysis (Quantibody Human Cytokine Array Q4000). Initial cutoff of differentially expressed proteins was performed by selecting the proteins with fold change > 2 for overexpressed proteins or < 0.5 for downregulated proteins and adjusted *p*-values of 0.05 for *edgeR* analysis using R software. Enriched pathways and biological significance were analyzed using the Gene Ontology tool *enrichGO* from the *clusterprofiler* version 3.15 package from Bioconductor (R software), and proteins were deemed significant if *p*-value < 0.01. The files to the raw data can be accessed through the Immunology Database and Analysis Portal (ImmPort) under study accession identifier SDY2196 [38].

### hBM-MSC immunopotency assay (IPA)

hBM-MSCs were co-cultured with human peripheral blood mononuclear cells (PBMCs) following the methods described by Bloom et al. [39]. Briefly, hBM-MSCs were seeded at a density of 2500 cells/well on topographical substrates attached to 96-well culture plates and were allowed to attach overnight. The next day, the media was changed with reduced serum formulation containing 2% FBS and cells were cultured for additional 24 h. PBMCs were labeled using 10 µM carboxyfluorescein succinimidyl ester—CFSE (ab113853, Abcam)—and seeded at a 1:0.5 ratio (PBMCs: hBM-MSCs) and co-cultured with hBM-MSCs for four days using RPMI-1640 containing 2% FBS, 1% NEAA, 1% P/S, anti-huCD-3 (2.5 ug/mL) and anti-huCD-28 (0.5ug/mL), except for the non-stimulated control that did not have CD-3 and CD-28. Afterward, PBMCs were harvested and their percentage of proliferation was analyzed using flow cytometry (BD Accuri™ C6 Plus).

*Long-term effect of the PS topography on the immunosuppressive capacity of hBM-MSCs:* hBM-MSCs were seeded on the topographical substrates and expanded for ten days under standard culture conditions. Afterward, the hBM-MSCs were detached from the topographical surfaces using trypsin EDTA 0.25% (59418C, Sigma) and subcultured into tissue culture treated flat surfaces. hBM-MSCs were allowed to attach overnight and then were co-culture with the PBMCs as described above.

### Statistics

Statistical analysis was performed using Graph Pad Prism 9.0 (GraphPad Software Inc, San Diego, USA) and the statistical software R version 4.2.0. The results are presented as the mean ± standard error of the mean (s.e.m), and differences between groups were performed using one-way and two-way ANOVA with a significance level (α) of 0.05 with Turkey’s comparison test.

## Results

### hBM-MSCs viability, morphology and metabolic activity on topographical cues in PS

**Fig. 1:**
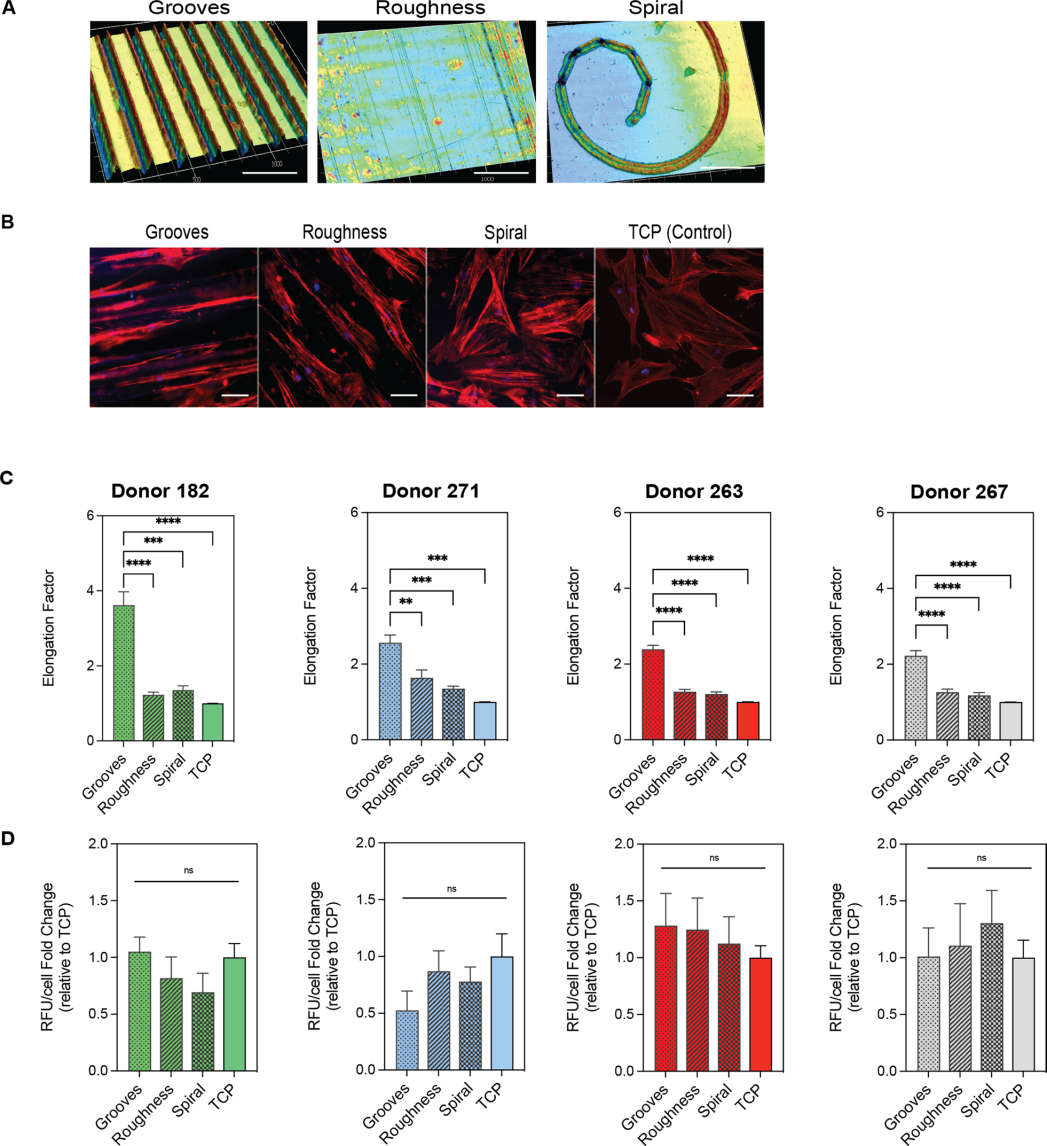
hBM-MSCs viability and morphology in the topographical substrates. **A** Laser confocal scanning 3D images of the grooves, roughness and spiral patterns PS films. Scale bar 50 µm. **B** Representative immunofluorescence images of hBM-MSCs cultured for 5 days in reduced serum (2% FBS) on the geometrical substrates and tissue culture plastic (TCP). The cytoskeleton of the cells was stained with actin red (red) and the nuclei with Hoechst (blue). Scale bar 100 µm. **C** Elongation of four different hBM-MSCs donors cultured in the topographic substrates. **D** PrestoBlue viability assay of four different hBM-MSCs donors quantified as relative fluorescence units per cell, relative to the control (TCP). Error bars depict the mean ± SEM of 3 independent experiments with *n* = 3–4 samples. One-way ANOVA. Asterisk (*) represent *p*-values < 0.05, (**) *p*-values < 0.01, (***) *p*-values < 0.001 and (****) *p*-values < 0.0001

hBM-MSCs were cultured on rough, spiral and grooved surfaces on polystyrene substrates (Fig. 1A–B) in culture media supplemented at a low serum concentration (2%). A low serum concentration was used to minimize the influence of exogenous biomolecules that can mask the effect of topographies on cell behavior [40]. Cell viability and morphology were measured as baseline metrics indicative of potential cell phenotype changes. Results show that the cell spreading on the polystyrene topographical surfaces was similar to standard tissue culture plastic (TCP), indicating successful hBM-MSCs attachment to the surfaces (Fig. 1B). As expected, cells cultured on anisotropic surfaces (grooves and roughness) were significantly more elongated than cells cultured on flat surfaces, being the cells cultured in grooved patterns at least twice as elongated as the flat surfaces, on average (Fig. 1C). This behavior was confirmed across four different hBM-MSCs donors. Interestingly, the elongation of the cells would take place along the edge of the ridge of the grooves and roughness, confirming the mechanosensitive capacity previously demonstrated in the literature for these cells [41–43]. Strikingly, donor 182 cell’s elongation was twofold more elongated than the rest of the donors. In this regard, cells from donor 182 cultured in grooved substrates had an increase of about 260% in elongation compared to TCP, also suggesting the variability in responses between donors, even if they are from the same tissue source. As expected, the viability of the cells cultured in the topographical substrates was not significantly different from flat surfaces (Fig. 1D).

**Fig. 2:**
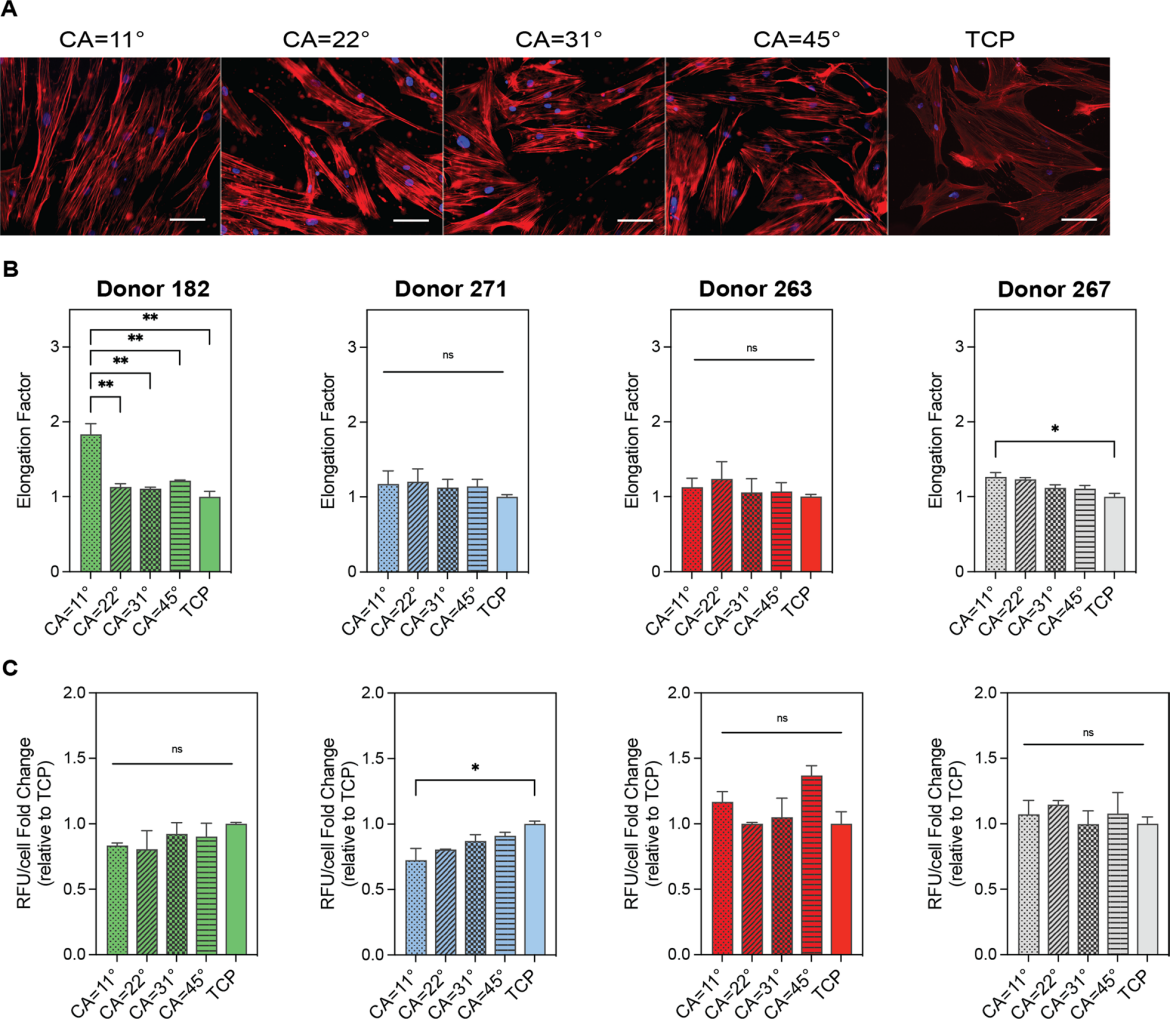
hBM-MSCs viability and morphology in substrates with different levels of wettability. **A** Representative immunofluorescence images of hBM-MSCs cultured for 5 days in reduced serum (2% FBS) on the substrates with levels of wettability and tissue culture plastic (TCP). The cytoskeleton of the cells was stained with actin red (red) and the nuclei with Hoechst (blue). Scale bar = 100 µm. **B** Elongation of four different hBM-MSCs donors cultured in the topographic substrates. **C** PrestoBlue viability assay of four different hBM-MSCs donors quantified as relative fluorescence units per cell, relative to the control (TCP). Error bars depict the mean ± SEM of 3 independent experiments with *n* = 3–4 samples. Asterisk (*) represent *p*-values < 0.05, (**) *p*-values < 0.01, (***) *p*-values < 0.001 and (****) *p*-values < 0.0001

PS substrates with surface contact angles of 11°, 22°, 31° and 45° were generated for these studies. The cytoskeleton organization of each cell donor was assessed by image-based analysis of cell morphology parameters of the actin cytoskeleton. Cell spreading was observed for all surface contact angles confirming cell attachment (Fig. 2A). Results in Fig. 2B showed a positive correlation between surface wettability levels and the elongation factor. Specifically, donor 182 displayed a twofold increase in elongation compared to the other donors on surfaces with a contact angle of 11°, suggesting that this donor is particularly more sensitive to topography stimuli than the others. As expected, cell viability was not significantly different to the standard TCP surface (Fig. 2C).

**Fig. 3:**
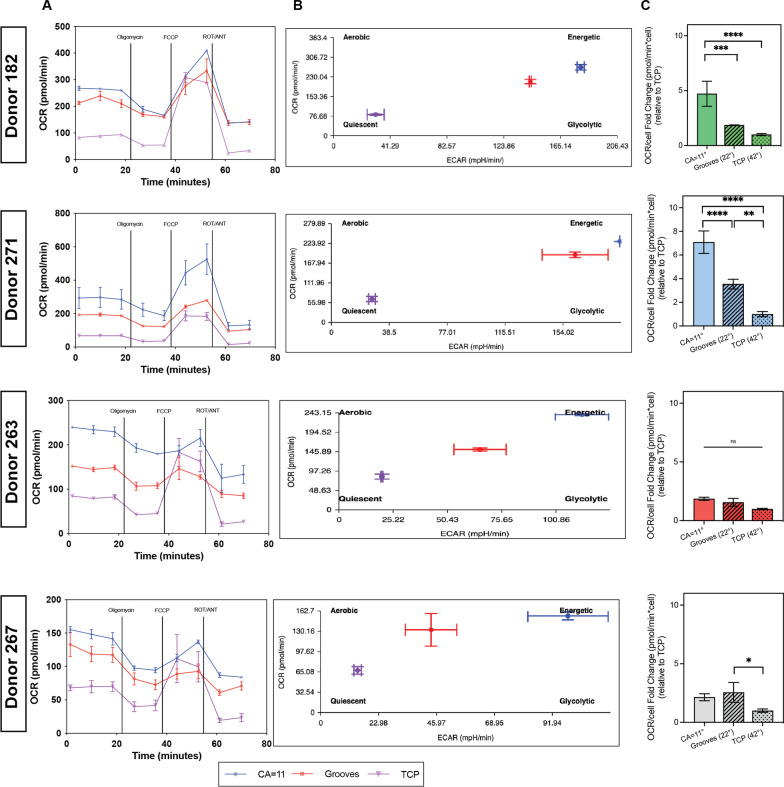
Metabolic activity of hBM-MSCs cultured in topographical substrates. **A** Metabolic profile of hBM-MSCs cultured in grooved and high-wettability substrates. Cells were cultured for 24 h in reduced serum media (2% FBS) and the OCR was evaluated with the Mito Stress Assay using the Agilent Seahorse XF technology. **B** Energy map showing metabolic phenotype of cells cultured in the topographical substrates. **C** OCR at basal level, relative to TCP control. Data are presented for the four hBM-MSCs donors 182, 271, 263 and 267 (top to bottom). Error bars depict the mean ± SEM of 3 independent experiments with *n* = 2 samples. Asterisk (*) represent *p*-values < 0.05, (**) *p*-values < 0.01, (***) *p*-values < 0.001 and (****) *p*-values < 0.0001

Previous studies have established that morphologic changes (depicted by cell elongation) and increased metabolic activity are independently correlated with enhanced immunosuppressive potency of mesenchymal stem cells [44–46]. To investigate the effect of enhanced cell elongation of PS topographies in the metabolic activity of hBM-MSCs, we accessed the oxygen consumption rate (OCR) to quantify mitochondrial respiration and the extracellular acidification rate (ECAR) as a measure of glycolysis on grooved micropatterns and surfaces of CA = 11° (high wettability). Results show that the oxygen consumption rate (OCR) on topographical substrates was significantly higher compared to TCP (Fig. 3C). hBM-MSCs from donors 182 and 271 cultured on CA = 11° surfaces displayed basal respiration rates four- to sixfold higher than cells cultured in TCP. The same trend was observed for the other two donors (263 and 267) but at a reduced magnitude. Cells cultured in topographical surfaces were consistently more energetic, meaning that the cells utilized the main two energy-producing pathways (oxidative phosphorylation and glycolysis), whereas cells cultured in flat surfaces exhibited a quiescent phenotype (cells were not energetic via either energy pathway), which is expected for cells cultured in low serum (Fig. 3B). The level of stimulation achieved by topographical cues was variable, where donors 182 and 271 are more sensitive than donors 263 and 267 to the physical stimuli. These results suggest that surfaces that promote cell elongation support a more energetic state in the hBM-MSCs.

### Surface topographies modulate the mechanosensing state and secretory profile of MSCs

**Fig. 4:**
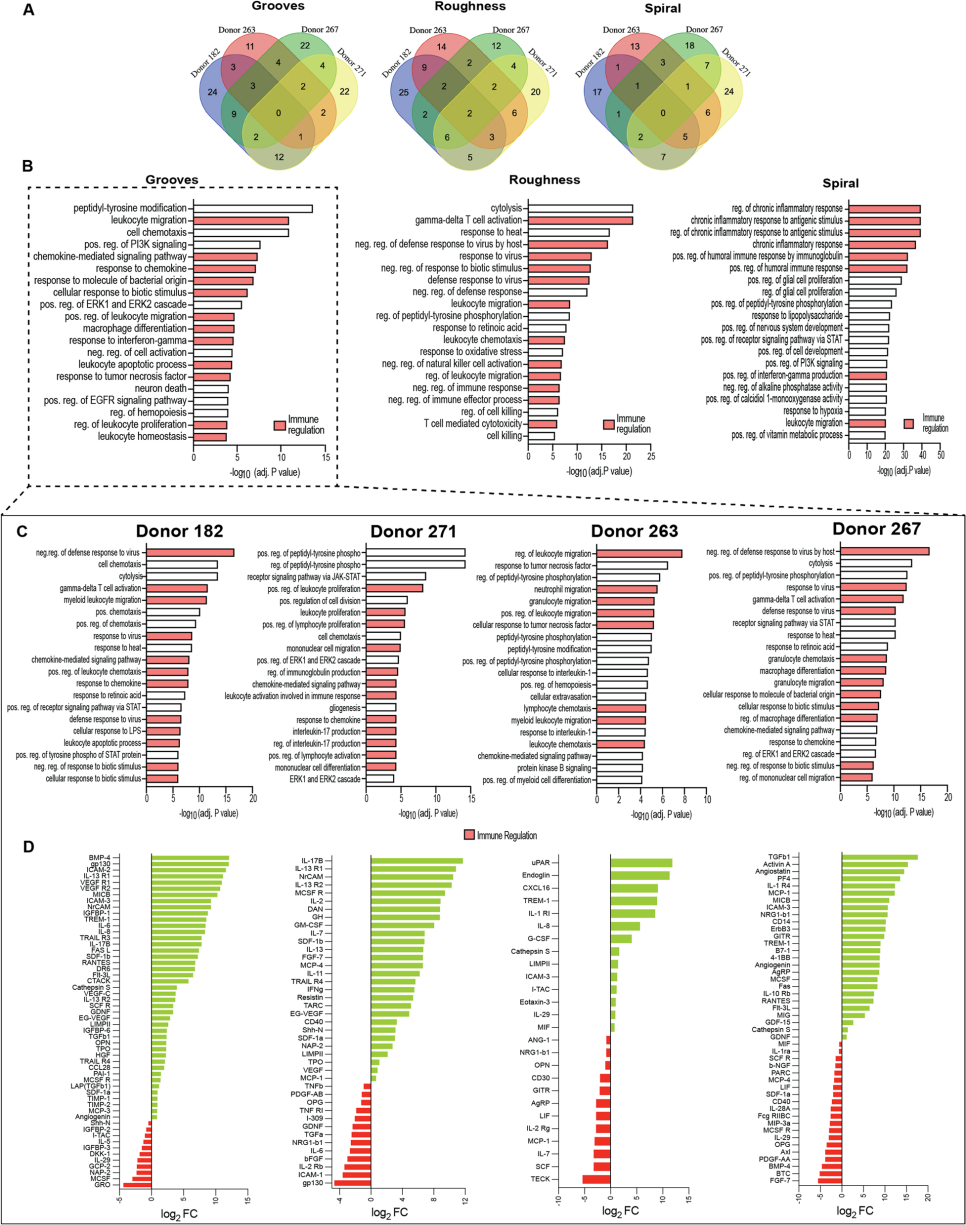
Protein secretion profile of hBM-MSCs cultured in topographical substrates. **A** Venn diagrams of differentially expressed proteins (fold change > 2 relative to TCP) of hBM-MCS from four different donors cultured in grooves, anisotropic roughness and spiral micropatterns. **B** GO top 20 enriched bioprocesses of hBM-MSCs cultured in grooves, anisotropic roughness and spiral micropatterns revealing enhancement in pathways related to immune regulation (adj. *p* < 0.01). **C** GO top 20 enriched bioprocesses of donors 182, 271, 263 and 267 (left to right) cultured in grooved surfaces (adj. *p* < 0.01). **D** Differentially upregulated (green) and downregulated (red) secreted proteins of donors 182, 271, 263 and 267 (left to right) cultured in grooved surfaces. Data depicts the log_2_ fold change (relative to the TCP control)

Given the enhanced metabolic activity of cells observed on surface topographies, cytokine secretion was examined in conditioned media to determine whether secretion is boosted by topographical stimuli. We found a broad amount of proteins enriched across the four donors in each of the surfaces. A total of 121 proteins were differentially expressed (DE) in the grooved surfaces compared to 114 and 106 in the anisotropic roughness and spiral substrates, respectively (Fig. 4A). As observed in previous results, the four donors are stimulated at different levels and the stimulation provided by the topographical cues is also expressed differently at the gene expression level across the donors. Only two proteins were found in common between the four donors in the hBM-MSCs cultured in anisotropic roughness, whereas no DE proteins were secreted in common between the donors cultured in grooves and spiral micropatterns. Gene ontology (GO) analysis concealed differences in secretory patterns at the bioprocess level. Approximately 40–60% of the top 20 bioprocesses were related to immune regulation and another few related to neural development (Fig. 4B). Grooved micropatterns showed the highest impact in terms of enriched pathways for immune regulation; thus, we proceeded to look further into individual donors. As expected, at least half of the DE proteins in the donors were related to immune regulation (Fig. 4C–D). Among the immunomodulatory cytokines secreted by MSCs across the four donors are the TGFβ family (*TGFβ1 *[47]*, BMP-4, Activin A*)*,* interleukins (*IL-6 *[48]*, IL-8, IL-10, IL-17B *[49]*, IL-13, IL-11, IL-2 *[50]*, IL-29 and IL-7 *[51])*,* chemokines (*MCP-1 *[52]*, MCP-3, MCP-4, SDF 1b/1a, RANTES, I-TAC and CCL28*), VEGF family (*VEGF-A, VEGF-C *[53]*)* and immunoglobulin family (*ICAM-3 *[54]) [55, 56]. Of these groups, molecules such as *TGFβ*, *IL-6*, *MCP-1*, *RANTES, VEGF* and *ICAM* have been identified in the literature as the most important immunomodulatory cytokines and are responsible for T-cells and other immune cells proliferation [57], inhibition of the apoptosis of neutrophils [58] and migration of immune cells [59]. In agreement with our previous observations in the cell energy state, donors 182 and 271 seemed to be more sensitive to the topographical stimuli as the amount of enhanced cytokines and bioprocesses related to immune regulation was superior relative to the other two donors.

Another fundamental component of the MSC secretome are the exosomes. Exosomes are secreted vesicles that play a key part in the therapeutic effects of the secretome of MSCs [60]. To further investigate the effect of topographical cues in exosome secretion, conditioned media from grooves and CA = 11° surfaces was analyzed for the presence of CD63 + exosomes. However, only donor 182 displayed enhanced secretion of exosomes compared to TCP (Additional file 2: Figure S2A-B). Violin plots were generated to recapitulate the donor-to-donor variability of the topographical stimuli and clearly, grooves and CA = 11° have higher variability response upon physical stimuli (Additional file 2: Figure S2C). Thus, although exosomes were not uniformly impacted across donors, the soluble factor data indicates that indeed enhanced metabolic activity on topographies correlates with a boost in the secretion of factors impacting bioprocesses associated with immune regulation.

**Fig. 5:**
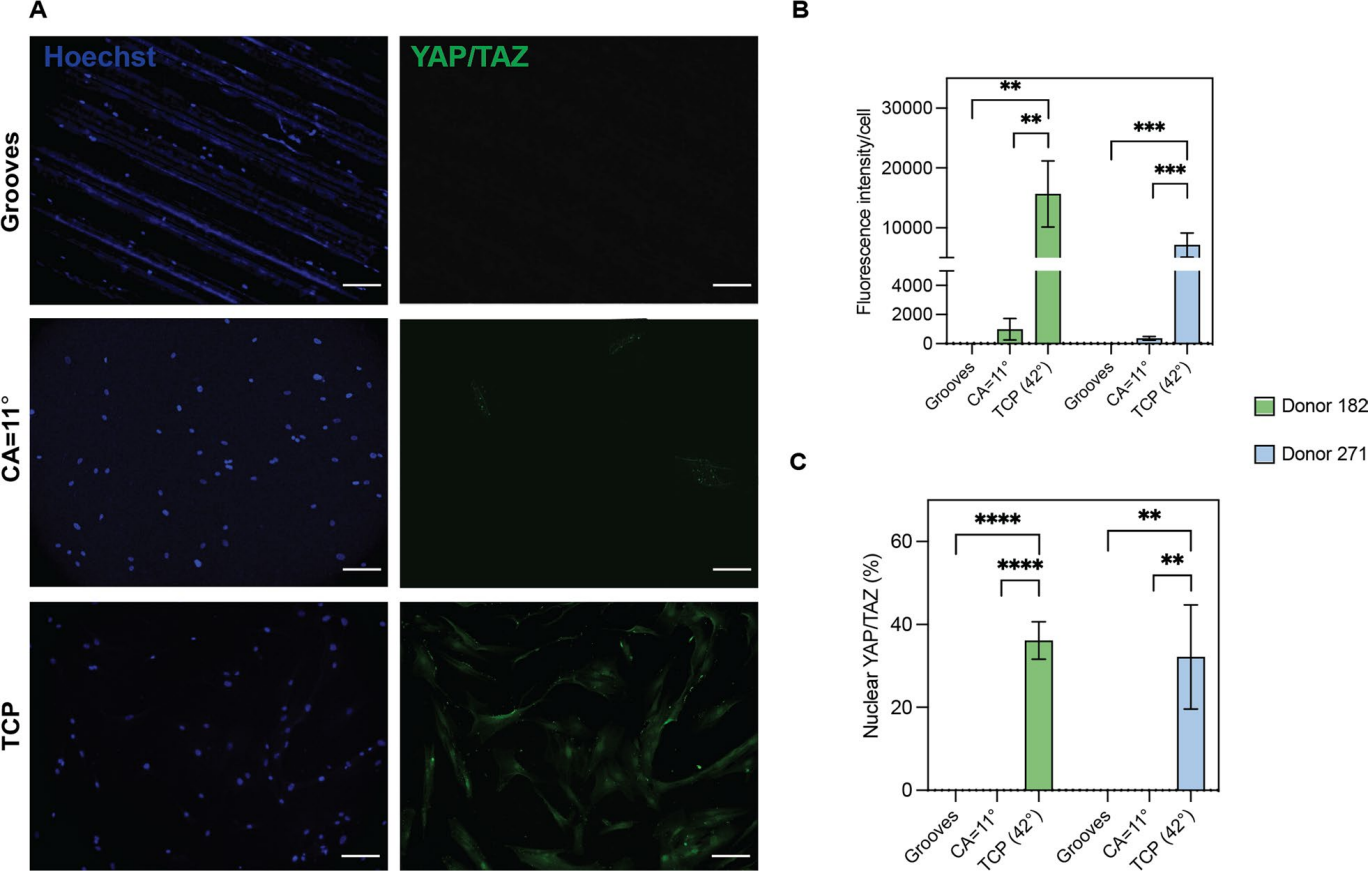
YAP/TAZ levels in hBM-MSCs cultured in topographical substrates. **A** Representative immunofluorescence images of hBM-MSCs donor 182 showing distinct distributions of YAP/TAZ (green) in the topographical surfaces. Scale bar = 100 µm. **B**–**C** Image-based analysis of YAP/TAZ expression levels in hBM-MSCs donors 182 and 271 cultured on each substrate. Data show the average total (**B**) and nuclear (**C**) levels for each donor. Error bars depict the mean ± SEM of 2 independent experiments with *n* = 27–65 quantified cells. Asterisk (*) represent *p*-values < 0.05, (**) *p*-values < 0.01, (***) *p*-values < 0.001 and (****) *p*-values < 0.0001

To confirm that our topographies are exerting mechanosensing signals in the cells, the expression of two well-known mechanotransducers, Yes-associated protein (YAP) and the transcriptional coactivator with PDZ-binding motif (TAZ), was examined [61, 62]. Results showed that the expression of total YAP/TAZ significantly decreased in grooves and flat surfaces with a CA = 11° compared to TCP. Specifically, YAP/TAZ was not detected in cells cultured on grooved surfaces, and low cytoplasmic levels were detected on CA = 11° surfaces (Fig. 5A–B). Furthermore, nuclear levels of YAP/TAZ were only detected in TCP (Fig. 5C), which indicates topographical-mediated changes in the mechanosensing of the cells. The modulation of YAP/TAZ levels was consistently seen in both donors 182 and 271, and suggest that the mechanical stimuli generated by the surface topographies is sufficient to modulate the energetic and secretome profile of cells.

### Immunosuppressive potency of hBM-MSCs is enhanced on topographical substrates

**Fig. 6:**
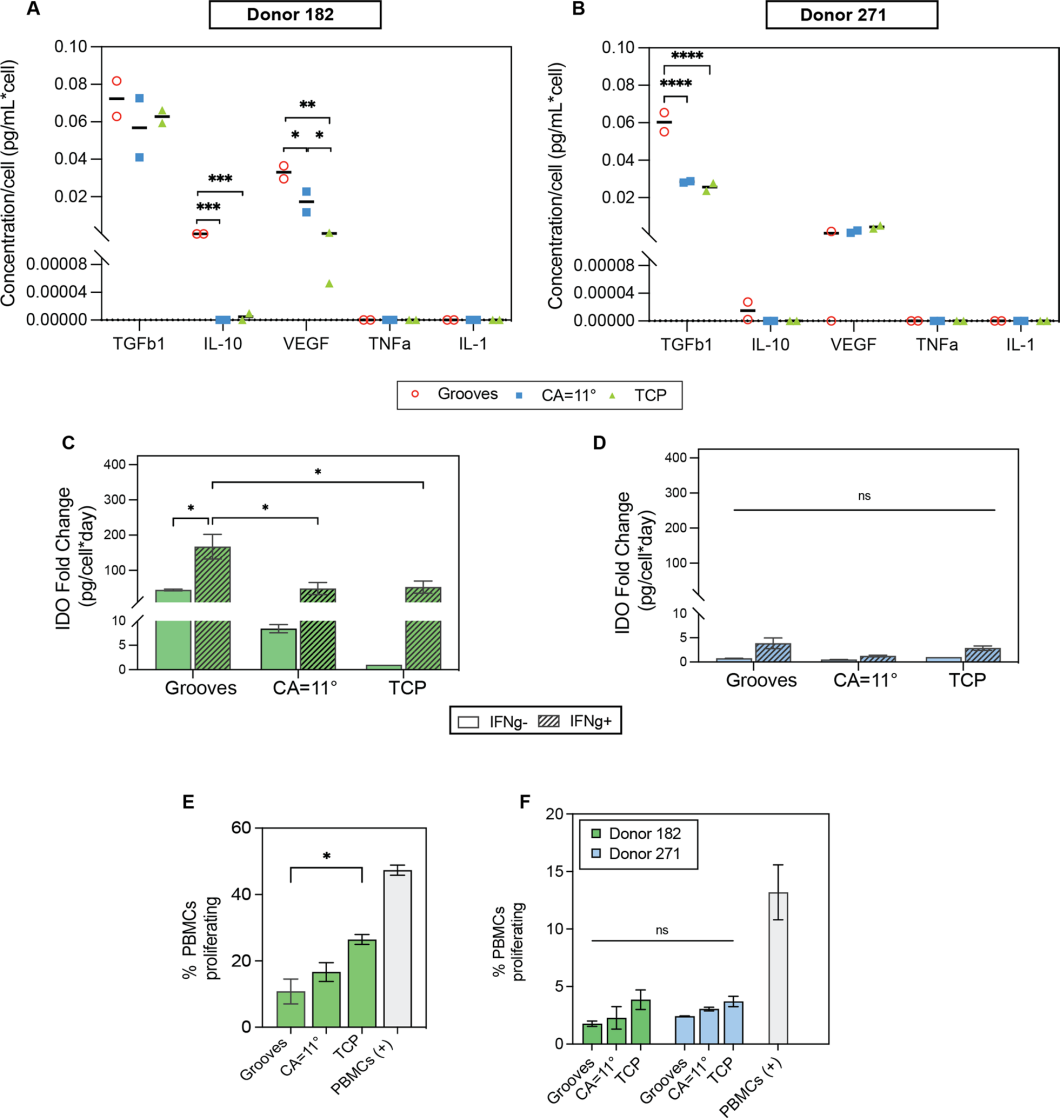
Immunosuppressive potency of hBM-MSCs cultured in topographical substrates. **A**–**D** Cytokine secretion of hBM-MSCs donors 182 and 271 cultured on topographical substrates. Cells were cultured for 5 days in reduced serum conditions (2% FBS) and quantification of target proteins was performed using enzyme-linked immunosorbent assays (ELISA), except for IDO **C**–**D** which was quantified via the kynurenine colorimetric assay and results are displayed as a fold relative to TCP. **E** Quantification of hBM-MSCs potency of donor 182 to suppress the proliferation of peripheral blood mononuclear cells (PBMCs); immune potency assay (IPA). Cells from donor 182 were seeded on the topographical substrates and co-cultured with PBMCs for 4 days in reduced serum conditions at a 1:0.5 ratio (PBMC:MSC). Proliferation of CFSE-stained PBMCs was measured using flow cytometry. **F** Evaluation of hBM-MSCs potency to suppress PBMCs proliferation after being cultured for 2 passages in the topographical substrates and then transferred to TCP surface and co-cultured with PBMCs as described above. Error bars depict the mean ± SEM of 2 independent experiments with *n* = 2 samples. Asterisk (*) represent *p*-values < 0.05, (**) *p*-values < 0.01, (***) *p*-values < 0.001 and (****) *p*-values < 0.0001

To examine the immunomodulatory potency of the secretome of hBM-MSCs on surface topographies where bioprocesses associated with immune regulation were found enriched, a quantitative analysis of selected biomolecules combined with functional assays was performed using donors 182 and 271. We measured the expression of TGFβ1, IDO, IL-10 and VEGF, cytokines established in the literature as mainly responsible for the immunomodulatory potency of MSCs [55]. Again, results showed that topographical substrates stimulate the secretion of cytokines at different levels in each hBM-MSCs donor, yet overall enhancement of the secretion of TGFβ1, IL-10 and VEGF-A was observed, especially in grooved micropatterns (Fig. 6A–B). Particularly, grooved substrates enhanced IL-10 expression over 35-fold in both donors compared to the TCP surface. In a similar manner, both topographical surfaces increased the secretion of VEGF in donor 182 above 44-fold and grooved surfaces doubled the expression of TGFβ1 in donor 271. The secretion of inflammatory cytokines TNF-ɑ and IL-1 were below the detection levels for both donors in all the surfaces. IDO secretion was also significantly enhanced in donor 182 cells cultured in the topographical surfaces and boosted upon stimulation with the inflammatory factor IFN-ɣ (Fig. 6C–D). Consistent with previous results, the secretome of donor 182 showed higher degree of sensitivity to topographical cues. Thus, collectively, results shown indicate that the secretion of anti-inflammatory factors is favored over inflammatory factors on the selected topographies supporting an immunosuppressive cell phenotype in hBM-MSCs.

The immunosuppressive potency of the secretome of hBM-MSCs was confirmed using a functional cell assay of growth suppression of peripheral blood mononuclear cells (PBMCs) in co-cultures. hBM-MSCs cultured on grooves and CA = 11° surfaces reduced the proliferation of PBMCs relative to flat surfaces, which agrees with previous results from the bioprocess secretome profiling (Fig. 6E–F). The proliferation of PBMCs dropped between 37 and 60% with hBM-MSCs cultured in the topographical substrates. Moreover, this enhanced immunosuppressive phenotype was still maintained in the hBM-MSCs from donors 182 and 271 after the cells were reseeded on TCP (Fig. 6F), which is in agreement with prior studies showing a mechanical memory retention of hBM-MSCs to the past physical culture environment [63, 64]. Consistently with our prior secretome data, grooved surfaces induced a more robust immunosuppressive stimuli where donor 182 immunosuppressive potency was superior to donor 271. Overall, the data strongly support that enhanced secretory capacity of hBM-MSCs can be stimulated by topographical cues and these stimuli lead the cells to a more immunosuppressive phenotype.

## Discussion

A major challenge in the use of in vitro environmental cues to influence MSCs behavior is the lack of studies correlating physical characteristics of substrates with cell function, limiting the development of strategies that effectively manipulate their therapeutic potency. Studies by Beijer et al. provided a good evaluation of how MSCs adapt to surface micropatterns, but did not specifically correlate the characteristics of the topographical surface with defined cell phenotypes [65]. Other studies use different materials to perform their studies on the effect of topographical cues in MSCs behavior; however, multiple questions have arisen on whether the observed effect is indeed due to the topographical cues or the biochemical composition [66]. In this work, we used in vitro physical cues on the culture surface of polystyrene, specifically microtopographies, to study their influence in the morphology, metabolism and secretory activity of hBM-MSCs. Overall, we found that topographical cues produce morphologic changes in the cells, depicted by cell elongation, where anisotropic topographies and surfaces with high wettability promoted elongation of the cells. Similarly, others have shown that changes in substrate geometry and roughness favored elongation and in turn multipotency markers and differentiation [67]. Our results agree with the previously reported mechanosensitivity of these cells, where anisotropic surfaces such as microgrooves and roughness did enhance hBM-MSCs elongation. Importantly, we showed that these topographies reconfigure the metabolic activity of hBM-MSCs toward an aerobic glycolysis state, boost the secretion of immunomodulatory factors and in turn, the immunosuppressive capacity of hBM-MSCs.

Surface wettability is a rather unexplored parameter in the culture of MSCs. Changes in surface wettability have been reported unfavorable for differentiated cells lines [68], yet our studies show that increased wettability is suitable for achieving enhanced elongation in hBM-MSCs. Similarly, few studies have shown that MSCs attach, proliferate and differentiate better in hydrophilic/wettable surfaces (water contact angles between 20° and 40°) versus hydrophobic/low-wettability surfaces (water contact angles between 40° and 90°) [69–71]; however, to the best of our knowledge, there are no reports that link morphology changes of MSCs with changes in surface wettability and specific functional changes in the cells such as the secretome and metabolic activity. Here, our results demonstrate that surface wettability does generate morphologic changes that will definitely have implications at the functional level. Nevertheless, even though previous studies have shown that cell elongation can be used as a predictor of MSCs immunosuppressive capacity and that surface topography indeed has a direct effect on cell morphology changes, our findings suggest that cell elongation should not be used solely as a morphologic fingerprint to predict the immunosuppressive potency of hBM-MSCs [44]. In fact, mitochondrial activity measurements and the secretome were more predictive of immunosuppressive potency. This observation is further supported by prior studies from Yao et al*.* and others correlating enhanced oxygen respiration rates with secretory function [72, 73].

MSCs reprogram their energy metabolism during differentiation, upon immune activation and changes in their microenvironment [74, 75]. Yet, most culture environment studies that measure metabolic activity on MSCs have been focused on biochemical stimulation rather than physical parameters. Although there is no specific data on MSCs, a study by Singh and collaborators showed that astrocytes cultured in micropatterned grooves enhanced their mitochondrial activity and these could represent progress in nervous system repair mechanisms [76]. Specific to MCSs, previous findings have shown that enhanced immunosuppressive potency of MSCs is directly related with a switch in their energy metabolism toward aerobic glycolysis [45, 72, 77]. Excitingly, in our study we found that hBM-MSCs reprogrammed their metabolism from a mostly senescent oxidative state to an energetic state using oxidative respiration and glycolysis when cultured in our topographical substrates. Consistent with this, our results also indicate that hBM-MSCs cultured in our topographical surfaces exhibited enhanced immunosuppressive capacity. Furthermore, we demonstrated that hBM-MSCs retained immunosuppressive memory, attributed to the exposure to the topographical substrates. Thus, these topographical cues can be used to stimulate MSCs anti-inflammatory potency in the short term but can also be seen as a way to generate cell populations with enhanced therapeutic capabilities without the need for licensing the cells, decreasing the overall costs of initial cell expansion.

Analysis of hBM-MSCs secretome indicated that topographical cues impacted the secretion of immune regulating factors. The topographical substrates examined enhanced the secretion of important immunomodulatory factors such as TGFb1, IL-10, VEGF and IDO, among others, and in turn their immunosuppressive potency. Our studies and others have previously demonstrated similar results, where surface topographies modulate MSC secretome and thus their therapeutic potential. Leuning et al. with their TopoChip described how surface topography can modulate the secretion of functional factors such as IL-6, HGF and SDF-1ɑ in bone marrow MSCs and kidney perivascular MCSs [78]. Also, other studies have demonstrated that microwell geometry (circular or squared) and microscale curvature affect IL-6 levels and promote the secretion of pro-angiogenic factors, respectively [79, 80]. Yet, some of these studies used adhesion biomolecules such as vitronectin and fibronectin, which have many disadvantages including increased initial culture costs and fast degradation over time, making them less appealing for cell manufacture applications [81]. Also, the use of integrin binding surface coatings can mask the overall contributions of the topographical cues to the observed changes in cell behavior. Our approach extends these observations by focusing on the physical stimuli of the topographical cues only and correlating secretome profiles with functional outcomes.

YAP/TAZ activation/deactivation, commonly regulated by the Hippo signaling pathway, have previously been reported to be affected by mechanical stimuli of the microenvironment [82]. In particular, many studies have demonstrated that the translocation of YAP/TAZ to the nucleus of cells is often mediated on stiffer substrates and increased surface rugosity [27, 83]. In our study, we observed decreased YAP/TAZ expression or no expression in the cytosol or the nuclei of cells cultured in our substrates compared to TCP. The reduction in YAP/TAZ may be a consequence of increased cytoskeletal tension in the topographies examined. For example, changes in surface elevation (topographical height) can be sensed by the cells and have been shown to modulate the effective modulus of elasticity of the surface. In fact, there is an inverse relationship between the height of a topographical feature and the stiffness [84]. Although we did not measure Hippo expression, our results suggest that changes in the elevation of our topographical substrates may translate in a decrease stiffness perceived by the cells and hence reduce the activity of the Hippo cascade and YAP/TAZ expression. The observed enrichment in immunomodulatory bioprocess may also be impacted by YAP/TAZ activity, as several studies have demonstrated that the lack or downregulation of the Hippo signaling is related to immune response and immunosuppression in MSCs [85, 86].

Donor variability in the responses of MSCs have been observed before, and this mainly attributed to the heterogeneity within MSCs populations [87, 88]. For example, changes in differentiation [89] potential of MSC cultured in tissue-engineered constructs and secretion of immunomodulatory factors [90] have been related to donor-specific variations. Donor-to-donor variability to topographical stimuli was not detected by changes in cell morphology and metabolic activity in grooves and high-wettability surfaces, but substantial differences were captured in the repertoire of secreted biomolecules. The topographical substrates did enhance the inherent secretory capabilities of the cells, but factors released differ from donor to donor. However, this donor-to-donor discrepancies can be minimized by performing statistical classification techniques such as clustering analysis. This approach groups factors at the bioprocesses level, which can be used to consolidate donor-to-donor differences to help identify clear paths to assess and score cell potency.

Despite the great potential of surface topographies to modulate cell behavior, a major difficulty is the microfabrication techniques. Many of these methods have high costs of investment, require specially trained personnel and long fabrication time [37]. Here, our study expands on the feasibility of razor printing, surface polishing and wettability to generate topographical substrates on PS films compatible with standard culture plates. We use our topographical array as an alternative to classical microfabrication methods (e.g., laser ablation, hot embossing or lithography), where our approach has fast prototyping time, requires low technical expertise, equipment investment and can be easily adapted into in vitro culture platforms for high-throughput analyses. Also, PS as raw material provides prolonged shelf storage life and maintains its physical properties during culture. This knowledge could be used to generate topographical features in microcarriers for upscaled culture systems to control MSCs secretome and enhance the yield of molecules of therapeutic interest. However, alternative microfabrication techniques (e.g., imprinting, lithography, 3D printing) to our razor printing approach will have to be employed to generate surfaces like the microgrooves in the microcarriers [91, 92]. Also, further considerations on how these topographies affect hBM-MSCs in a 3D microenvironment such as a bioreactor will have to be evaluated.

## Conclusions

The goal of this study was to evaluate the impact of surface topographies in the secretory activity of BM-hMSC. We highlighted grooved micropatterns and high level of surface wettability as key topographical cues in supporting enhanced secretion and immunosuppressive phenotype in BM-hMSCs. Overall, this work demonstrates the potential of topographical cues as a culture strategy to enhance the secretory capacity of MSCs.

### Supplementary Information

**Additional file 1**. **Figure S1**: Surface wettability characterization and stability.

**Additional file 2.** **Figure S2**: Exosome secretion is enhanced in topographical surfaces.

## Data Availability

The datasets generated and/or analyzed during the current study are available in the Immunology Database and Analysis Portal (ImmPort) repository, https://www.immport.org/shared/study/SDY2196.

## Abbreviations

CFSE: 5,6-Carboxyfluorescein succinimidyl ester
DE: Differentially expressed
DMEM: Dulbecco’s modified eagle's medium
EV: Extracellular vesicles
FBS: Fetal bovine serum
GO: Gene ontology
hBM-MSC: Human bone marrow-derived mesenchymal stem cells
IDO: Indoleamine 2,3-dioxygenase
IFN-ɣ: Interferon gamma
IL: Interleukin
MSC: Mesenchymal stem cells
OCR: Oxygen consumption rate
PBMC: Peripheral blood mononuclear cells
PBS: Phosphate-buffered saline
PS: Polystyrene
SEM: Standard error of the mean
TCP: Tissue culture plastic
TGFb1: Transforming growth factor beta 1
TNF-ɑ: Tumor necrosis factor alpha
VEGF: Vascular endothelial growth factor
