# Supplementary figures and images for: Substrate topographies modulate the secretory activity of human bone marrow mesenchymal stem cells
Source: Stem Cell Res Ther. 2023 Aug 21;14:208. doi: 10.1186/s13287-023-03450-0 (PMC10441765; doi:10.1186/s13287-023-03450-0)

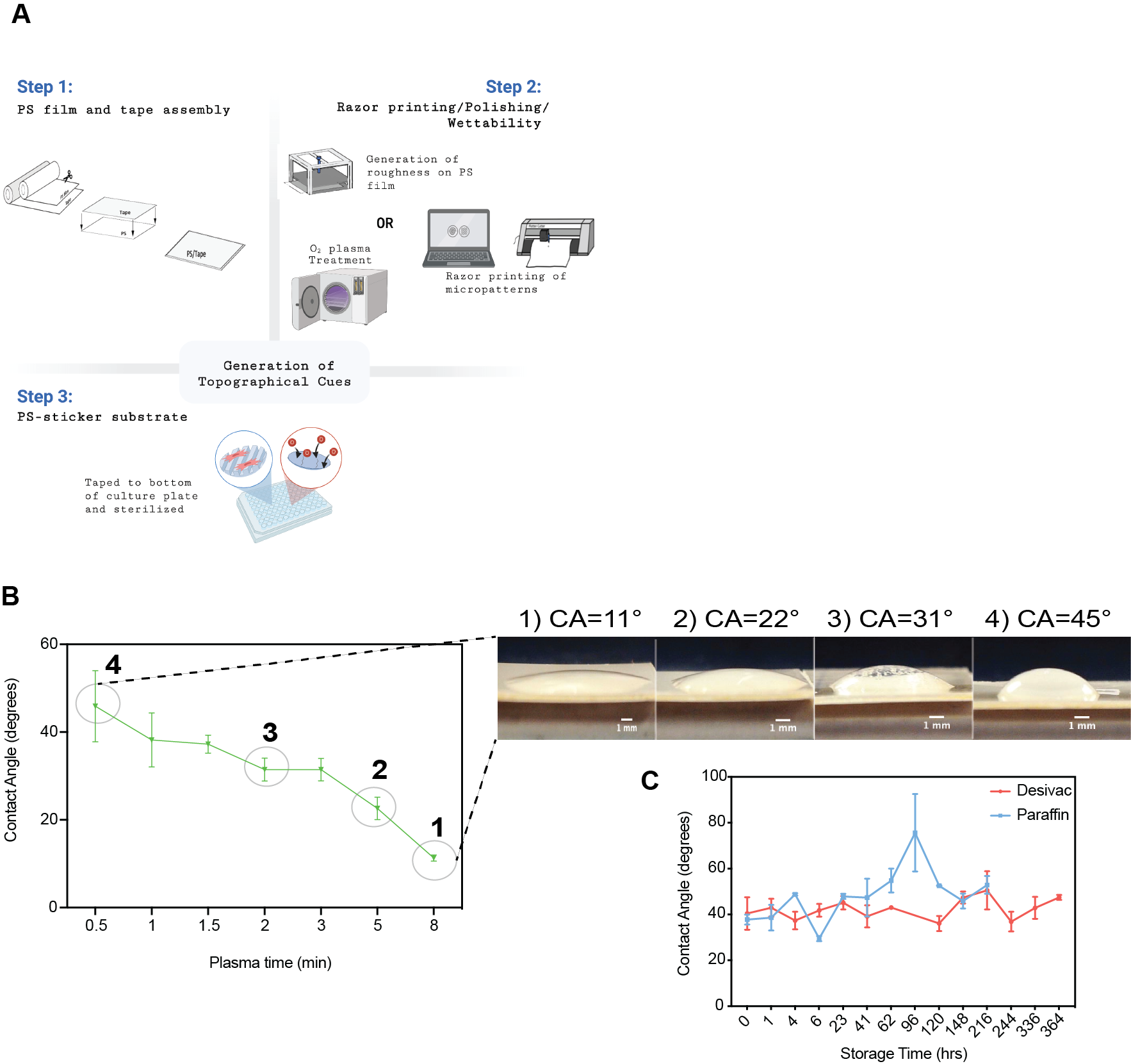

Supplement: Supplementary file 1 — Additional file 1. Figure S1: Surface wettability characterization and stability. [file 13287_2023_3450_MOESM1_ESM.png]

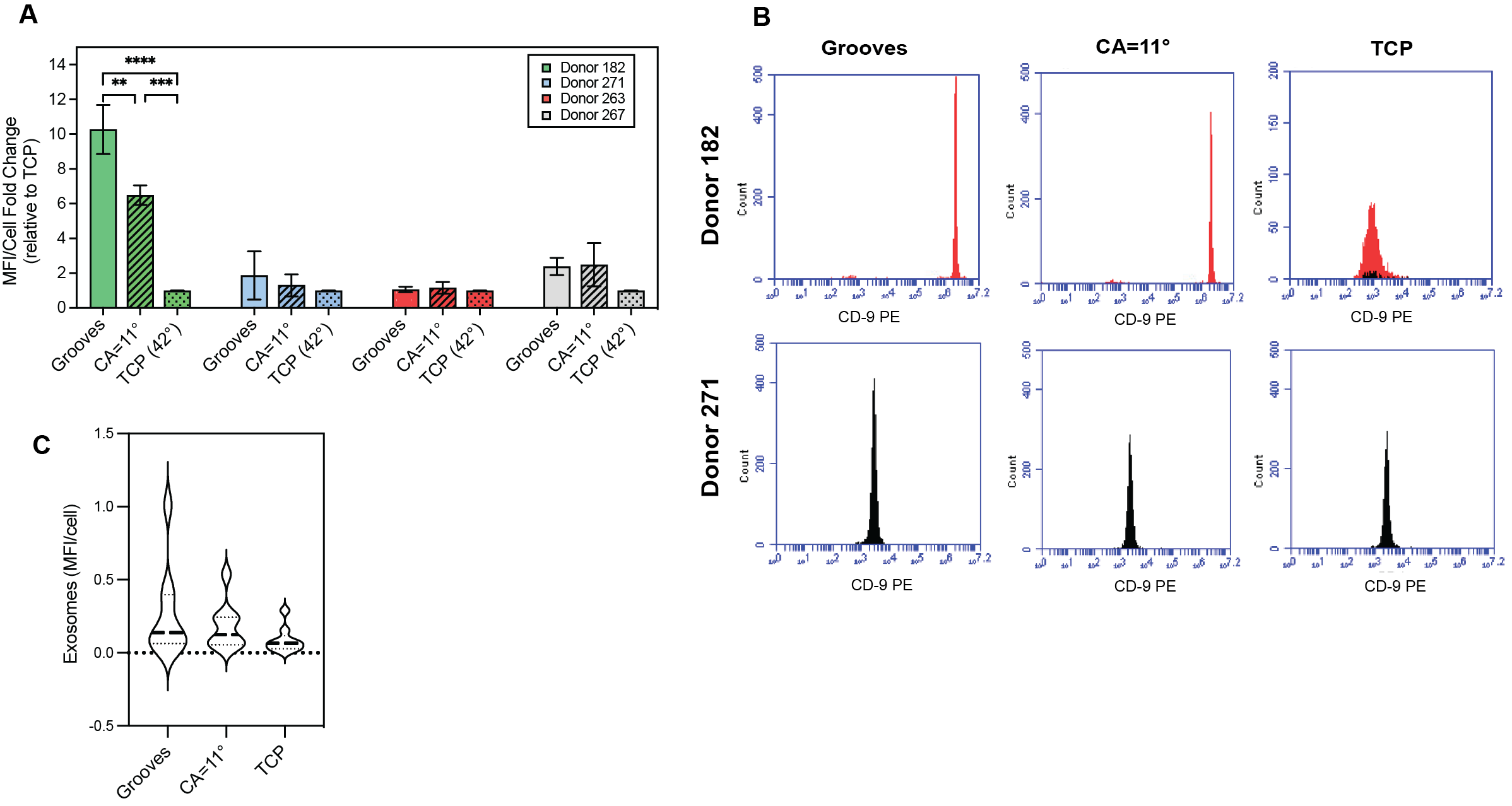

Supplement: Supplementary file 2 — Additional file 2. Figure S2: Exosome secretion is enhanced in topographical surfaces. [file 13287_2023_3450_MOESM2_ESM.png]
